# Inflammatory Adipokines, High Molecular Weight Adiponectin, and Insulin Resistance: A Population-Based Survey in Prepubertal Schoolchildren

**DOI:** 10.1371/journal.pone.0017264

**Published:** 2011-02-18

**Authors:** Giuseppe Murdolo, Bettina Nowotny, Federica Celi, Miranda Donati, Vittorio Bini, Francesco Papi, Gabi Gornitzka, Serena Castellani, Michael Roden, Adriano Falorni, Christian Herder, Alberto Falorni

**Affiliations:** 1 Section of Internal Medicine, Endocrine and Metabolic Sciences, Department of Internal Medicine, Perugia University, Perugia, Italy; 2 Institute for Clinical Diabetology, German Diabetes Center, Leibniz Center for Diabetes Research at Heinrich Heine University, Düsseldorf, Germany; 3 Section of Pediatrics, Department of Surgical Sciences and Public Health, Perugia University, Perugia, Italy; 4 Department of Metabolic Diseases, University Clinics, Heinrich Heine University Düsseldorf, Düsseldorf, Germany; Boston University, United States of America

## Abstract

**Background:**

The aim of this study was to investigate sex differences and associations of high molecular weight (HMW) adiponectin, leptin and proinflammatory adipokines, individually or in combinations, with adiposity and insulin resistance (IR) measures in prepubertal childhood.

**Methodology:**

We studied 305 prepubertal children (boys/girls: 144/161; Tanner stage 1; age: 5-13 yr), included in a cohort of 44,231 adolescents who participated in an extensive Italian school-based survey. According to Cole's criteria, 105 individuals were lean (L; boys/girls: 59/46), 60 overweight (OW; boys/girls: 32/28) and 140 obese (OB; boys/girls: 70/70). Measurements comprised total and HMW adiponectin, leptin, as well as a panel of proinflammatory adipokines/chemokines associated with diabetes risk.

**Principal Findings:**

Leptin-, and the leptin-to-HMW adiponectin ratio (L/HMW)-, increased progressively (p<0.0001) from L to OW to OB boys and girls. When compared with L peers, OW and OB girls exhibited lower (p<0.001) HMW adiponectin levels, while in boys the HMW multimers did not differ significantly across the BMI-stratified groups. OB girls displayed higher (p<0.05) IL-8, IL-18, monocyte chemoattractant protein-1 (MCP-1) and soluble intercellular adhesion molecule-1 levels (sICAM-1) than L girls, whereas increased macrophage migration inhibitory factor (MIF) concentrations in OB vs OW boys were seen. HMW adiponectin (negatively), leptin or inflammatory markers (positively) correlated with adiposity and IR measures. In multivariate models, leptin represented a strong and independent determinant of HOMA-IR (R^2^ 0.378; p<0.01). Adjustment for age, BMI*_z-score_*, lipids and inflammatory mediators abolished the association between leptin and HOMA-IR in boys, while in girls leptin remained still a significant predictor of IR (R^2^ 0.513; p<0.01). Finally, in both sexes, the joint effect of the L/HMW did not improve the prediction of basal IR as compared with leptin levels alone, which were mainly explained by the BMI*_z-score._*

**Conclusions:**

In prepubertal children, leptin emerges as a sex-independent discrimination marker of adiposity degree and as a useful, sex-associated predictor of the systemic insulin resistance.

## Introduction

Accumulating evidence indicates that chronic low-grade inflammation independently predicts the development of type 2 diabetes (T2D) and coronary heart disease (CHD) [1]. The emerging role of adipose tissue as a bioactive endocrine organ secreting an ever increasing number of mediators, collectively known as adipokines, supports the concept that fat tissue *per se* is a strong determinant of systemic inflammation and a culprit underlying both the insulin resistance (IR) and vascular risk [2].

Thus far, the alarming growth of childhood obesity is leading to a concomitant rise of the so-called “cardiometabolic syndrome” even in youth, explaining the relationship between higher BMI in adolescence and greater disease risk in adulthood [3]. It is also becoming clear that, as early as in childhood, obesity poses an inflammatory environment both in the adipose tissue [4], [5], [6] and in the circulation, as reflected by increased concentration of inflammatory adipokines (ie, cytokines and chemokines) and reduced levels of adiponectin, an insulin-sensitizing, anti-inflammatory adipose-derived mediator [7], [8], [9]. Adiponectin is also known to be secreted in different oligomeric forms, with the high molecular weight (HMW) multimers being the most biologically active [9]. Indeed, in obese adolescents, the impaired secretion of the HMW oligomers, rather than deficiency of total adiponectin, emerges as a stronger predictor of IR and incipient atherosclerosis [10], [11], [12]. Moreover, circumstantial data in obese young children suggest that elevated concentrations of “prototypical” inflammatory cytokines (ie, IL-6 and TNF-α), adiponectin deficiency and hyperleptinemia, which is also a sequel to chronic inflammation [13], may coexist [7], [14], [15]. Altogether, these observations strengthen the concept that the dysfunctional pattern of adipokines in the bloodstream is initiated in, and sustained by, the “inflamed” fat [2]. However, the role of different adipose-derived mediators (chemokines), individually or in combination, as independent prediction markers of impaired glucose homeostasis and future diabetes development in prepubertal age is still controversial [8], [16], [17].

In this scenario, the characterization of a comprehensive profile of adipokines in adolescence appears of considerable interest for identification of “at-risk” individuals. Accordingly, in adults, the joint effect of leptin and adiponectin, as ascertained by the leptin-to-adiponectin ratio, has been proposed as a more reliable predictor of IR and vascular risk than measurement of adiponectin and leptin alone [18], [19], [20]. Since the enlarged/inflamed adipocytes secrete more leptin and less adiponectin, especially the HMW isomers [9], one may argue that the ratio of leptin-to-HMW adiponectin (L/HMW) could serve as a useful indicator of adipocyte dysfunction also before puberty begins.

As yet, in prepubertal childhood, the interrelations between HMW adiponectin, leptin and different inflammatory adipokines, as well as the effect modification by sex and adiposity have not been ascertained in detail [11], [21], [22]. The overall aims of this study were therefore to investigate sex differences and associations of HMW adiponectin, leptin and proinflammatory adipokines with adiposity and insulin resistance measures in prepubertal age. To achieve this goal, we studied a cohort of prepubertal children included in an extensive school-based survey, and characterized the clinical phenotype, the endocrine and metabolic profile, as well as a panel of adipokines/chemokines associated with adiposity and glucose homeostasis. We postulated that, since gonadal steroids may affect adiponectin oligomers distribution and circulating leptin levels, partly explaining sex differences in association between inflammatory markers and diabetes risk [23], a dysregulated repertoire of these molecules in prepubertal age would reflect the “quality” of individual adipose cells as well as their association with insulin-resistance better than in adulthood [4], [11], [21], [22], [24], [25], [26].

## Methods

### Participants

We studied 305 Italian children (boys/girls:144/161) aged 5–13 years, included in a cohort of 44,231 adolescents who participated in an extensive school-based project on growth conducted in the provinces of Perugia, Terni and Rieti of central Italy. The survey design and methods have been previously described in detail [27], [28]. In the present study we enrolled only children who fulfilled the following eligibility criteria: 1) prepubertal age (Tanner stage 1); 2) absence of acute or chronic inflammatory illness; 3) lack of diabetes mellitus, primary hyperlipidemia, hypertension and obesity due to endocrine diseases; and, 4) no current regular medications.

Written informed consent was obtained from the parents of the children before their participation in the study, which has been approved by the Ethics Committee of the Umbria Region and carried out in accordance with the principles of the Helsinki Declaration.

### Anthropometric measurements

Anthropometric data included height, body weight, body mass index (BMI), blood pressure, waist and hip circumferences, and the waist-to-hip ratio (WHR) [27], [28], [29], [30]. Body fat mass was estimated by bioelectrical impedance analysis (BIA, Akern S.r.l, Florence, Italy). Pubertal stage was classified according to Tanner, whereas the standard deviation score of BMI (BMI*_z-score_*) was calculated based on Cole's method on central Italy L (lambda) M (mu) S (sigma) reference values [28], [31]. The LMS method summarizes the distribution of BMI at each age in terms of three smooth curves by its median (M), coefficient of variation (S) and the Box-Cox power (L), required to transform raw data to a standard normal distribution. Child overweight (OW) or obesity (OB) were categorized according to age- and sex-specific international cut-off points for BMI, identified for the percentiles corresponding to BMIs of 25 (OW) and 30 (OB) kg/m^2^ at 18 years, respectively [31]. Finally, systolic (SBP) and diastolic (DBP) blood pressure values were standardized (Z-score) to the subjects' sex, age and height, in order to provide a precise diagnostic classification according to body size [32].

### Biochemical analyses

Blood samples were collected during routine analysis, after overnight fasting. Subjects were requested to maintain their usual diet 8-10 days before testing. After separation, sera aliquots were immediately stored at −70°C until analyzed. Follicle stimulating hormone (FSH) and luteinizing hormone (LH) were determined by immunoradiometric (IRMA) assay (CT Immunotec; Turin; Italy). Total testosterone (DRG, Milan, Italy) and dehydroepiandrosterone-sulphate (DHEA-S) (Radim, Rome, Italy) were assessed by radioimmunoassay (RIA), while free-testosterone and estradiol concentrations were evaluated by immunuoenzymatic assay (DiaMetra, Milan, Italy). Fasting plasma insulin was measured by RIA (Bouty, Cassina de' Pecchi, Milan, Italy), with intra- and inter-assay coefficients of variation (CV) <10%. Homeostasis model assessment of insulin resistance (HOMA-IR) was calculated as follows: fasting insulin (µU/ml) × fasting glucose (mmol/l)/22.5. Total adiponectin levels were analysed by RIA (Linco Research, St. Charles, MO, USA). HMW adiponectin oligomers were quantified by an enzyme-linked immunosorbent assay (ELISA) from ALPCO Diagnostics (Salem, NH, USA), according to the manufacturer's instructions. Under our experimental conditions, the intra- and inter-assay CV were both <5%. Finally, the serum concentration of leptin, interleukin (IL)-8, IL-18, monocyte chemoattractant protein-1 (MCP-1), regulated on activation, normal T-cell expressed and secreted (RANTES), macrophage migration inhibitory factor (MIF), soluble intercellular adhesion molecule-1 (sICAM-1), interferon-γ-inducible protein 10 (IP-10) and resistin were simultaneously measured by bead-based multiplex assay using a Luminex 100 analyser (Luminex Corporation, Austin, TX, USA), as described [1], [14]. Fluorescent xMAP COOH microspheres were purchased from Luminex. Recombinant proteins were obtained from MBL (Nagoya, Japan; IL-18), R&D Systems (Wiesbaden, Germany; MCP-1), the National Institute for Biological Standards and Controls (Potters Bar, UK; IL-8), and BD Biosciences (Heidelberg, Germany; IP-10). Antibody pairs were purchased from MBL (IL-18), R&D Systems (MCP-1, IL-8), and BD Biosciences (IP-10). Assay characteristics for the measurement of leptin, IL-8, IL-18, MCP-1, MIF, IP-10, resistin, RANTES and sICAM-1 were as follows: intra-assay CVs were all <5%; inter-assay CVs, 14, 18, 17, 16, 11, 21, 13, 20 and 16%, respectively; limits of detection, 9.8, 0.6, 0.6, 2.4, 4.9, 0.6, 2.4 pg/ml and 2.4 and 9.8 ng/ml, respectively. The rationale behind the selection of this specific panel of inflammatory molecules in our Luminex platform was that: 1) growing data underscore the role of chemokines as modulators of macrophage infiltration into adipose tissue and predictors of diabetes risk [1], [33], [34], [35]; and, 2) no prior study evaluated sex differences and associations of these mediators with adiposity and IR measures in prepubertal age.

### Statistical analysis

Categorized comparisons for sex- and BMI-stratified groups were analyzed by one-way ANOVA and Kruskal-Wallis test with appropriate post-hoc corrections (Bonferroni's, Tukey's and Kruskal-Wallis Z). Accordingly, for each response variable, the main effects of sex and BMI, as well as their interaction, were further ascertained by general linear models (factorial design). Univariate correlations were assessed by Spearman rho (*r_s_*) and partial correlation, while multivariate relationships were evaluated using multiple regression models. The explanatory variables entered the regression models according to the significance of their correlation coefficients in bivariate analysis.

Before analysis, non-normally distributed data were appropriately transformed (Box-Cox transformation) to better approximate the Gaussian distribution (Kolmogorov-Smirnov test). Data are shown as mean±SD or median (25^th^; 75^th^ percentile) for parametric and non parametric variables, respectively. P-values <0.05 were regarded as statistically significant. Statistical analysis was performed using Predictive Analytic Software release 17.0.2 (SPSS Inc., Chicago, IL, USA).

## Results

### Subjects Characteristics

Participants' characteristics are outlined in Table 1. When compared with lean (L), OB children exhibited increased abdominal fat distribution, higher fasting insulin, HOMA-IR and triglycerides levels, differences which were partly independent from sex. Indeed, fasting insulin and HOMA-IR, which showed a significant (p = 0.01) sex-by-BMI-group interaction, were increasing with adiposity in girls, whereas in boys the OW and the L children displayed similar HOMA-IR values. Within either sex, no significant group differences for blood pressure *z-score* or sex steroids were found. OB girls, however, displayed higher FSH concentrations, when compared with OB boys, and increased DHEA-S levels, in comparison with L peers.

**Table 1:** Characteristics of the study population stratified by sex and BMI.

	Boys			Girls		
	Lean (L)	Overweight (OW)	Obese (OB)	Lean (L)	Overweight (OW)	Obese (OB)
n.	59	32	70	46	28	70
Age (yrs)	9.2±2	9.9±1.3 1	8.6±1.1	8.6±1.8	8.8±1.6	9.3±1.3
BMI (kg/m^2^)	16.0 (15.1–17.6)^2^	22.0 (20.9–22.7)^3^	25.7 (24.5–27.6)	15.1 (14.2–16.2)^4^	21.3 (19.5–22.7) 3	25.1 (23.8–26.8)
BMI_z-score_	−0.45±0.7 2	1.2±0.2 3	2.0±0.3	−0.64±0.8 4	1.2±0.2^5^	2.1±0.4
Fat mass (kg)	5.3 (3.7–7.3)^2^	10.8 (9.5–14.8) 3	18.0 (14.1–22.8)	4.4 (3.4–5.6)^4^	12.3 (9.0–14.01) 3	15.7 (13.2–18.8)
Waist circumference (cm)	58.2 (53.2–61.0) 2	73.5 (68.2–76.0)^5^	81.0 (76.0–87.3)	54.0 (51.2–57.5) 4	68.7 (66.6–74.5) 3	78.0 (73.0–82.0)
WHR	0.85±0.04^6^	0.88±0.04	0.92±0.07	0.85±0.04^6^	0.91±0.15^7^	0.88±0.05
Fasting glucose (mmol/l)	4.5±0.5	4.4±0.5	4.4±0.4	4.2±0.6	4.3±0.4	4.4±0.4
Fasting insulin (pmol/l)	40.9 (28.7–56.7)	44.8 (37.5–58.8) 8	66.0 (50.2–89.0) 5	38.7 (21.5–52.2)^8^	59.2 (43.1–103.0)^9^	68.9 (52.4–97.6)^5^
HOMA-IR	1.1 (0.8–1.7)	1.2 (1.0–1.5)	1.7 (1.3–2.3) 10	1.0 (0.5–1.5)^8^	1.6 (1.2–1.4)^11^	1.8 (1.4–2.6)^9^
Total cholesterol (mmol/l)	4.2 (3.9–4.9)	4.4 (3.8–4.8)	4.1 (3.8–4.6)	4.6 (3.8–5.3)	4.2 (3.8–4.6)	4.2 (3.8–4.8)
HDL-C (mmol/l)	1.2 (1.0–1.3)	1.1 (1.0–1.5)	1.2 (0.9–1.4)	1.2 (1.1–1.4)	1.2 (1.0–1.4)	1.1 (0.9–1.3)
Triglycerides (mmol/l)	0.6 (0.5–0.8) 12	0.7 (0.5–0.8)^13^	0.8 (0.6–1.1)	0.7 (0.6–0.8)^6^	0.8 (0.5–1.1)	0.8 (0.7–1.3)
SBP_z-score_	−0.070±0.912	0.426±0.922	0.157±1.020	0.001±0.882	0.132±0.889	0.317±0.987
DBP_z-score_	0.295±0.672	0.356±0.779	0.547±0.712	0.249±0.864	0.206±0.751	0.357±0.687
FSH (IU/L)	1.3 (0.6–2.0)	0.7 (0.6–1.6)	0.5 (0.1–1.0)^14^	1.6 (0.6–2.5)	1.5 (0.7–2.9)	1.0 (0.4–2.5)
LH (IU/L)	0.2 (0.1–0.8) 11	0.3 (0.1–0.8)	0.1 (0.1; 0.4)	0.1 (0.1–0.2)	0.2 (0.1–0.5)	0.2 (0.2–0.3)
Total testosterone (nmol/l)	1.5 (0.7–1.7)	0.7 (0.6–1.6)	0.7 (0.7–1.0)	0.7 (0.7–1.0)	0.7 (0.7–1.3)	0.7 (0.7–1.0)
Free-testosterone (pmol/l)	2.2 (1.4–3.5)	2.1 (1.4–3.5)	2.4 (1.6–3.8)	2.4 (1.6–3.8)	3.1 (1.9–3.6)	2.4 (1.7–3.5)
Estradiol (pmol/l)	NA	NA	NA	73.6±32.6	72.9±32.6	64.3±31.4
DHEA-S (µmol/l)	1.0 (0.8–2.5)	1.8 (1.1–2.9)	1.6 (0.8–2.8)	0.9 (0.4–1.3)^7^	0.9 (0.6–1.5)	1.3 (0.8–2.0)

**^1^**p<0.001 vs L and OB girls;

**^2^**p<0.0001 vs OW and OB boys and vs OW and OB girls;

**^3^**p<0.0001 vs L and OB boys and vs L and OB girls;

**^4^**p<0.0001 vs OW and OB girls and vs OW and OB boys;

**^5^**p<0.0001 vs L and OB boys and vs L girls;

**^6^**p<0.0001 vs OB within the gender;

**^7^**p<0.0001 vs OB girls and vs OW and OB boys;

**^8^**p<0.001 vs OW and OB girls and vs OB boys;

**^9^**p<0.0001 vs L boys and girls;

**^10^**p<0.0001 vs L and OW boys and vs L girls;

**^11^**p<0.05 vs L girls;

**^12^**p<0.001 vs OB boys and girls;

**^13^**p<0.0001 vs OB girls;

**^14^**p<0.0001 vs L boys and vs L, OW and OB girls; NA: not assessed.

### Adiponectin and leptin

**Figure 1:**
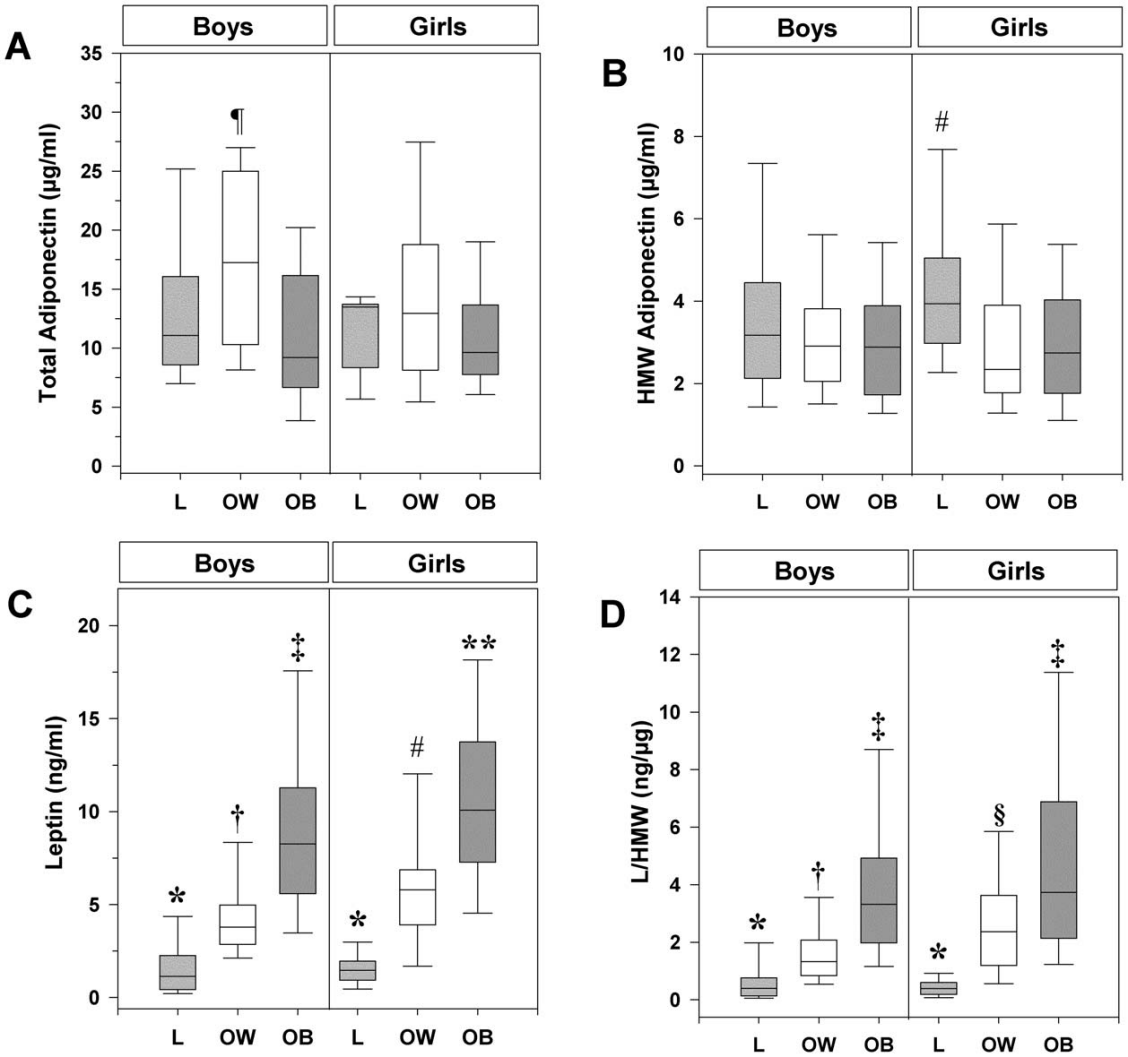
Box-whisker plots showing the levels of total and high molecular weight (HMW) adiponectin (panels A-B), leptin (panel C), and the leptin-to-HMW adiponectin ratio (L/HMW) in the BMI-categorized groups, according to sex. Boxes represent median (line in the middle of the boxes) and interquartile ranges (25^th^ and 75^th^ percentile; lines at the bottom and the top of the boxes, respectively). Error bars are 5^th^ and 95^th^ percentiles. Differences between groups were analysed by Kruskal-Wallis test with Bonferroni's post-hoc correction. L: lean; OW: overweight; OB: obese; ^¶^p = 0.001 vs OB boys and OB girls; ^#^p<0.001 vs OW and OB girls and vs OB boys; *p<0.0001 vs OW and OB boys and vs OW and OB girls; ^†^p<0.0001 vs L and OB boys and vs L and OB girls; ^‡^p<0.0001 vs L and OW boys and vs L girls; **p<0.0001 vs L and OW boys and girls; ^§^p<0.0001 vs L girls and boys.

In categorized comparisons across the groups, total and HMW adiponectin showed a different sex distribution pattern (Figure 1, panels A and B). In L children, while total adiponectin levels were comparable in both sexes, the HMW multimers tended to be higher in girls [3.9 (3.0; 5.0) µg/mL] than in boys [3.2 (2.1; 4.4) µg/mL]. As compared with L peers, both OB and OW girls exhibited similar total, but reduced (p<0.001) HMW adiponectin concentrations. In boys, although OW and OB subjects displayed HMW adiponectin levels similar to those of OW and OB girls, respectively, the HMW forms did not differ across the BMI-categorized groups. Moreover, total adiponectin concentrations were increased (p<0.01) in OW as compared with OB boys.

In both sexes, leptin and L/HMW (Figure 1, panels C and D) were increasingly higher with adiposity and differed significantly (p<0.0001) between L and OW, L and OB, as well as OW and OB individuals. Notably, neither leptin, nor adiponectin (total and HMW) as well as the ratio L/HMW showed significant sex-by-BMI-group interaction, implicating that at the same BMI category the response variable behaved the same when sex varied.

Overall, these data suggest that, while in L prepubertal children HMW adiponectin may exhibit a tendency toward a sexual dimorphism, leptin emerges as sex-independent discrimination marker of adiposity degree.

### Chemokine and Cytokine Concentrations

The concentrations of inflammatory chemokines and cytokines are shown in Table 2. In girls, OB subjects presented higher (p<0.05) IL-8, IL-18, MCP-1 and sICAM-1 levels, when compared with L peers. In boys, only MIF concentrations were significantly higher (p<0.05) in OB than in OW controls (Table 2). No consistent group changes were seen for RANTES, IP-10 and resistin serum levels. Interestingly, none of the above immune markers differed significantly between sexes within the same BMI-stratified group, and no consistent sex-by-BMI group interactions were seen.

**Table 2:** Cytokine and chemokine concentrations.

	Boys			Girls		
	Lean (L)	Overweight (OW)	Obese (OB)	Lean (L)	Overweight (OW)	Obese (OB)
n.	59	32	70	46	28	70
IL-8 (pg/ml)	4.1 (1.9–7.7)	4.8 (2.7–7.0)	5.0 (2.5–10.4)	4.6 (2.7–6.3)	3.4 (2.3–5.3)	5.3 (3.6–9.9)^1^
IL-18 (pg/ml)	241.3 (170.9–320.9)	188.4 (136.9–276.2)	200.1 (149.2–265.5)	182.6 (121.6–262.9)	178.1 (144.7–242.9)	240.6 (159.7–334.5) 1
MCP-1 (pg/ml)	688.7 (490.9–1058.9)	785.3 (611.7–987.9)	866.6 (601–1146.5)	543.7 (419.7–809.6)	781.1 (471.9–1038.4)	821.4 (677.7–1051.9) 1
RANTES (ng/ml)	138.6 (101.5–219.8)	143.1 (89.7–200.3)	166.8 (134.2–219.6)	132.3 (99.9–232.9)	163.7 (135.7–230.2)	185.1 (123.5–232.6)
MIF (pg/ml)	3756 (3221–4562)	3617 (3141–4043)^2^	4190 (3525–4784) 3	3893 (3244–5047)	3927 (3484–4639)	4332 (3585–5046)
sICAM-1 (ng/ml)	32.1 (29.0–38.4)	30.9 (26.1–37.0)	33.2 (28.0–40.0)	31.6 (26.5–36.4)	33.2 (28.6–43.1)	34.2 (29.2–40.1)^1^
IP-10 (pg/ml)	166.6 (131.7–259.1)	178.2 (130.9–241.1)	177.3 (145.8–221.2)	168.2 (125.9–245.0)	176.4 (129.5–276.6)	193.1 (159.2–256.3)
Resistin (pg/ml)	1701 (1276–1872)	1644 (1273–2017)	1728 (1412–2095)	1541 (1270–2140)	1539 (1320–2091)	1884 (1476–2168)

**^1^**p<0.05 vs L girls;

**^2^**p<0.05 vs OB girls;

**^3^**p<0.05 vs OW boys.

Altogether, these data confirm the lack of a universal upregulation of the immune response also in prepubertal obesity [14], implicating that factors other than sex may modulate the circulating levels of these mediators at this early age.

### Adipokines, Adiposity and Insulin Resistance

In order to ascertain the interrelationships between HMW adiponectin, leptin and inflammatory cytokines/chemokines, individually or in combinations, with sex, adiposity or IR, we performed correlation analyses and constructed multivariate models in the combined study population and within each sex.

First, in both sexes, we found no significant association of leptin, HMW adiponectin or inflammatory molecules with gonadal steroids (supplementary Table S1). However, HMW adiponectin (negatively), as well as both leptin and L/HMW (positively) correlated with adiposity and IR measures, triglycerides and sICAM-1 (supplementary Table S2). Interestingly, leptin and HMW correlated in a reciprocal manner with each other and with the inflammatory markers (supplementary Table S3). Also, the inflammatory cytokines/chemokines displayed numerous positive correlations with each other and with HOMA-IR (supplementary Table S3). Adjustment for BMI*_z-score_* almost eliminated the correlations of leptin and HMW adiponectin with the immune mediators, whereas reciprocal associations between the inflammatory markers with each other or IR measures remained partly unaffected (supplementary Table S4). These data suggest that adiposity explains, at least in part, the associations between HMW adiponectin, leptin or the inflammatory mediators on the one hand and impairment of glucose homeostasis on the other, while correlations among immune markers appear obesity-independent.

Second, multivariate regression models were constructed to predict HMW adiponectin levels (Table 3). As expected, fasting insulin was a predictor of adiponectin multimers, explaining alone about 5% of their variance (model 1). Adjustment for BMI*_z-score_* (model 2) attenuated this interaction, which became insignificant when lipids (models 3), or lipids, leptin and sICAM-1 entered the model (model 4). In the full model (model 5), however, BMI*_z-score_* was the strongest determinant of HMW adiponectin, and along with triglycerides it accounted for additional 5% of its variance. Moreover, in the prediction of HMW adiponectin, there were no statistically significant interactions between sex and the explanatory variables (all interactions p>0.05) (data not shown).

**Table 3:** Multiple regression models for the prediction of the high molecular weight adiponectin (HMW) (dependent variable) in the combined study population.

Model	Independent Variable(s)	β	*p-value*	Model R^2^
**1**	Fasting insulin	−0.221	<0.0001	0.049
**2**	Fasting insulin	−0.142	0.037	0.068
	BMI*_z-score_*	−0.160	0.019	
**3**	Fasting insulin	−0.100	0.151	0.093
	BMI*_z-score_*	−0.128	0.063	
	Triglycerides	−0.112	0.078	
	HDL	−0.122	0.041	
**4**	Fasting insulin	−0.127	0.085	0.102
	BMI*_z-score_*	−0.230	0.017	
	Triglycerides	−0.135	0.039	
	HDL	−0.130	0.099	
	Leptin	0.159	0.121	
	sICAM-1	−0.028	0.652	
**5**	Fasting insulin	−0.123	0.098	0.106
	BMI*_z-score_*	−0.225	0.027	
	Triglycerides	−0.144	0.030	
	HDL	0.108	0.085	
	Leptin	0.152	0.180	
	sICAM-1	−0.033	0.603	
	Age	0.012	0.867	
	Sex*	0.060	0.353	

*Sex entered in regression model as “dummy” variable (0 = girls; 1 =  boys).

β-coefficients, *p-*values and determination coefficients of regression models (R^2^) are given.

Third, we explored whether leptin, HMW adiponectin or the L/HMW might have explained the systemic IR as estimated by HOMA-IR. In the combined study population (Table 4), multivariate analysis indicated that each explanatory variable alone (model 1) was a significant predictor of HOMA-IR, while HMW adiponectin lost its significance after adjustments for age, BMI*_z-score_* and lipids (model 2), or when sex and inflammatory markers entered the regression model (model 3). In contrast, leptin represented a strong and independent determinant of HOMA-IR. However, we found statistically significant (p<0.05) interaction between sex and leptin. Thus, multivariate analyses for HOMA-IR were also stratified within each sex (Table 5). Of note, leptin remained a strong and independent predictor of basal IR in girls, while in boys its contribution became insignificant after adjustments for age, BMI*_z-score_* and lipids (model 2), or when the inflammatory markers entered in the model (model 3). Interestingly, in all the models, the joint effect of L/HMW did not improve the HOMA-IR prediction as compared with leptin alone, and the inflammatory mediators did not meaningfully contribute to changes in the outcome in a substantial way.

**Table 4:** Multiple regression models for the prediction of the basal insulin resistance as ascertained by HOMA-IR (dependent variable) in the total study population.

Independent variables									
Model	Leptin			HMW			L/HMW		
	β	*p-value*	Model R^2^	β	*p-value*	Model R^2^	β	*p-value*	Model R^2^
1*	0.551	<0.0001	0.304	−0.206	0.001	0.04	0.548	<0.0001	0.300
2†	0.326	<0.0001	0.352	−0.058	*NS*	0.323	0.306	<0.0001	0.355
3‡	0.291	<0.01	0.378	−0.055	*NS*	0.357	0.272	<0.01	0.381

*Unadjusted model.

† Model adjusted for age, BMI*_z-score_*, triglycerides and HDL-Cholesterol.

‡ Additional adjustment for sex, sICAM-1, IL-8, MCP-1, RANTES, MIF and IP-10. Sex entered in regression model as “dummy” variable (0 = girls; 1 = boys). NS, statistically not significant. β-coefficients and *p*-values for independent variables as well as determination coefficients of regression models (R^2^) are given.

**Table 5:** Multiple regression models for the prediction of the basal insulin resistance as ascertained by HOMA-IR (dependent variable) in boys and girls.

Boys									
Independent variables									
	Leptin			HMW			L/HMW		
Model	β	*p-value*	Model R^2^	β	*p-value*	Model R^2^	β	*p-value*	Model R^2^
1*	0.471	<0.0001	0.222	−0.124	*NS*	0.015	0.465	<0.0001	0.217
2†	0.193	*NS*	0.264	−0.04	*NS*	0.256	0.204	*NS*	0.269
3‡	0.167	*NS*	0.308	−0.057	*NS*	0.303	0.196	*NS*	0.314

Girls									
Independent variables									
	Leptin			HMW			L/HMW		
Model	β	*p-value*	Model R^2^	β	*p-value*	Model R^2^	β	*p-value*	Model R^2^
1*	0.627	<0.0001	0.393	−0.265	<0.01	0.070	0.606	<0.0001	0.368
2†	0.49	<0.0001	0.454	−0.058	*NS*	0.392	0.351	<0.01	0.434
3‡	0.415	<0.01	0.513	−0.044	*NS*	0.47	0.304	<0.01	0.500

*Unadjusted model.

† Model adjusted for age, BMI*_z-score_*, triglycerides and HDL-Cholesterol.

‡ Additional adjustment for sICAM-1, IL-8, MCP-1, RANTES, MIF and IP-10. *NS*, statistically not significant. β-coefficients and *p*-values for independent variables as well as determination coefficients of regression models (R^2^) are given.

Finally, multivariate models indicated the BMI*_z-score_* as a strong and independent determinant of leptin levels (Table 6), and, along with HOMA-IR and triglycerides, it accounted for ∼70% of its variance. Again, we failed to find statistically significant interactions between sex and the explanatory variables associated with leptin prediction (all interaction p>0.05). Accordingly, the multivariable models stratified within each sex basically recapitulated the results observed in the combined study population (supplementary Tables S5 and S6).

**Table 6:** Multiple regression models for the prediction of leptin (dependent variable) in the combined study population.

Model	Independentvariable(s)	β	*p-value*	Model R^2^
**1**	BMI*_z-score_*	0.779	<0.0001	0.606
**2**	BMI*_z-score_*	0.636	<0.0001	0.667
	HOMA-IR	0.192	<0.0001	
	Triglycerides	0.160	<0.0001	
	LDL	−0.045	*NS*	
**3**	BMI*_z-score_*	0.641	<0.0001	0.671
	HOMA-IR	0.194	<0.0001	
	Triglycerides	0.153	<0.0001	
	LDL	−0.044	*NS*	
	HMW	0.054	*NS*	
	MCP-1	0.003	*NS*	
	RANTES	0.054	*NS*	
	MIF	−0.035	*NS*	
	Sex*	−0.071	0.051	

*Sex entered in regression model as “dummy” variable (0 = girls; 1 =  boys). NS, statistically not significant; β-coefficients, *p-*values and determination coefficients of regression models (R^2^) are given.

Altogether, these findings circumstantiate the concept that, in prepubertal age, adiposity may concur with altered lipid profile to the impairment of HMW adiponectin, which appears partly independent from low-grade inflammation. Finally, in this cohort of prepubertal children, we found that leptin, at least in girls, was an independent determinant of the basal insulin resistance, and, independently from sex, it emerged as a stronger contributor of HOMA-IR than HMW adiponectin.

## Discussion

This study shows that, already in prepubertal age, obesity is linked with hyperleptinemia, impaired HMW adiponectin levels, as well as alterations of specific immune mediators with minor sex-associated differences. Notably, in this very young population, leptin, rather than HMW adiponectin, emerges as a sex-independent discrimination marker of adiposity degree, as well as a sex-related predictor of basal insulin resistance. Finally, also in prepubertal obesity, we confirm the lack of a universal upregulation of the immune response.

In obese children, persistent low-grade inflammation appears to increase the metabolic risk in later life. The importance to investigate adipose-derived mediators during the developmental stages of overweight and obesity allows to identify at what age and degree of adiposity these pathological events may occur. Our current work describes the combined associations of obesity, IR and sex on a cluster of adipokines in prepubertal children. The characterization of a wide panel of adipose-derived mediators, rather than measurement of single molecules only, appears of considerable importance in youth since current conventional risk factors may not be sensitive enough to detect high-risk individuals, and the prognostic information provided may be different to those in older obese subjects.

As yet, most of the studies on children analyzed a limited number of adipose-produced mediators, and comparative analyses for sex and pubertal stage were not always stratified over wide BMI ranges [11], [21], [22], [24]. Here we focused on prepubertal children in their early phase of fat gain, making confounding effects of co-morbidities or gonadal steroids unlikely. The first main finding was that, independently from sex, hyperleptinemia and, to a lesser extent, decreased HMW adiponectin, characterizes prepubertal obesity.

Among the adipokines, adiponectin and leptin are unique hormones because these pleiotropic mediators are almost exclusively secreted by the adipocytes, and their systemic levels reflect the degree of fat accrual in a reciprocal manner [9], [16]. Adiponectin, however, circulates in low- (LMW), middle- (MMW) and high-molecular weight (HMW) complexes, which exert different biological functions [9]. In this regard, already in obese adolescents, the selective downregulation of the HMW multimers has been shown to reflect metabolic and vascular abnormalities better than total adiponectin levels [10], [11]. Prospectively, in adults, hypoadiponectinemia, decreased HMW adiponectin and increased leptin concentrations were differently reported as independent predictors of T2D development and, less consistently, of CHD events [8], [9], [13], [26], [36]. Notably, in our study, adiposity-induced variations of leptin and, at least in female individuals, changes of HMW adiponectin concentrations, were of the same magnitude as those that predicted diabetes development in adulthood, suggesting an early effect of these molecules on the regulation of glucose homeostasis [8], [13], [18]. Thus, leptin upregulation along with decreased HMW adiponectin subfractions may both have a relevant impact for future disease risk in this subset of obese, otherwise healthy, prepubertal children.

As long as at this age consistent effects of gonadal steroids appear unlikely, different reasons may underlie the dysfunctional profile of these adipokines. First, the lack of interconversion of the adiponectin complexes in the bloodstream highlights the importance of the adipocyte secretory pathway in determining the circulating pattern of adiponectin isoforms [9]. In adult males, we have recently demonstrated that adipocyte enlargement and/or IR, rather than body fat mass or testosterone, appear prerequisites for the impaired cellular secretion of HMW adiponectin complexes [37]. Accordingly, in prepubertal children, intracellular content of adiponectin in subcutaneous adipocytes was reported to be reduced with increasing BMI*_z-score_* [6]. Our findings partly extend these results and implicate reduced HMW adiponectin and increased leptin levels as early indicators of adipocyte enlargement and cell dysfunction. The opposite associations between HMW and leptin with adiposity measures, along with the observation that BMI*_z-score_* was the strongest determinant of both leptin and HMW multimers, partly support such a concept.

Second, since hyperinsulinemia or IR may either induce hyperleptinemia or promote the selective downregulation of the HMW adiponectin forms [9], [38], a different degree of insulin sensitivity in the adipose cells may well have contributed to the impaired secretion of these adipokines. Accordingly, it has been demonstrated that total adiponectin concentrations are more closely related to variations in insulin-mediated glucose disposal than to fat mass expansion [39]. In our study, we showed that the associations of fasting insulin (Table 3) or HOMA-IR (supplementary Tables S3 and S4) with HMW adiponectin were attenuated or became insignificant after adjustments for BMI*_z-score_* or lipids, respectively. Thus, the association of HMW adiponectin with IR appears partly mediated by adiposity. In contrast, leptin, which was also largely explained by adiposity (Table 6), represented an independent and stronger predictor of the systemic IR as compared with HMW or the combinational effect of L/HMW (Table 4). The demonstration that in adipocytes adiponectin and leptin are secreted through distinct trafficking pathways [41], which appear modulated by insulin in different ways [38], may partly explain these findings, substantiating the hypothesis that derangements of glucose homeostasis develop at an early preclinical stage as a result of adipocyte dysfunction/enlargement [5], [6]. On the other hand, it has recently been postulated that, in obese prepubertal children, HMW adiponectin shows closer relationship with improvement of glucose homeostasis after weight loss, rather than with body fat content [42]. Nonetheless, we have previously demonstrated that, in obese children and adolescents, the degree of hyperleptinemia was a reliable predictor of responsiveness to an educational program of weight excess reduction [29], [30]. It seems thus plausible that, in prepubertal age, leptin may provide prognostic information (ie, prediction of weight-excess reduction) different to that of HMW adiponectin (ie, improvement of glucose homeostasis after weight loss).

Third, although HMW adiponectin, leptin and inflammatory markers appear to some extent interdependent, the addition of leptin and sICAM-1 as “inflammation-related” confounders did not improve the prediction of HMW adiponectin, fostering the concept that, although mutually antagonistic, low-grade inflammation and HMW adiponectin may reflect two distinct features of the immune response, as previously postulated [43].

In adolescents, various and controversial studies addressed the sex-associated variability of total adiponectin and leptin [11], [21], [22], [24], [25], while information on HMW is scarce. In our cohort, when BMI-categorized groups were analyzed, a tendency toward a sex-dimorphic pattern for HMW adiponectin in lean individuals was found, whereas leptin allowed a sex-independent discrimination of adiposity degree. However, for both leptin and HMW adiponectin we did not find significant sex-by-BMI-group interaction, implicating the lack of consistent changes of the response variable when, at the same BMI category, sex varied. Even more, sex did not affect the prediction of BMI*_z-score_* on HMW adiponectin (Table 3) and leptin (supplementary Tables S5 and S6). Hitherto, although two population-based surveys found higher total adiponectin levels in girls than in boys even in prepubertal age, consistent with our results other authors did not [21], [24], [25]. These discrepancies may largely reflect the lack of comparisons across differentially categorized groups. Here, we adopted Cole's criteria [31] and incorporated a broad spectrum of clinical phenotypes, making thus the occurrence of group misclassification unlikely. It is conceivable, and remains to be shown, that inherent characteristics of adipocyte cellular function as well as variations in molecular degradation or clearance rate may influence the levels of these mediators in lean prepubertal children [9].

On the other hand, sex represented an “effect modifier” in the interaction between leptin and basal IR. Our observation that leptin was an independent determinant of HOMA-IR in girls, but not in boys (Table 5), is however in line with the notion that, in adulthood, inflammation appears more strongly associated with IR and diabetes risk in females than in males [23], [40]. As far as in our study sex differences regarding body composition and systemic gonadal steroids levels (ie, testosterone) were not apparent, such sex-associated differences may have several explanations. Different degrees of adjustment in the multivariable models (ie, larger sex-differences according to the number of confounders) as well as chance (reduced sample size) might both have played a role. Moreover, although we can reasonably exclude the effect of ethnicity, the influence of different baseline risk factors of IR cannot be ruled out.

Turning to the other major finding of this study, childhood obesity is not characterized by a generalized immune activation, but rather a differential upregulation of specific chemokines/cytokines with minor sex differences [14]. Obese girls, indeed, displayed higher IL-8, IL-18, MCP-1 and sICAM-1 levels, while in boys adiposity was linked with increased MIF concentrations. The lack of consistent sex-by-BMI-group interaction, as well as the absence of significant correlations between gonadal steroids and all of the above immune mediators (supplementary Table S1), reasonably indicates that factors other than sex (ie., nutritional habits, physical activity) may partly explain these differences. Although no prior study has evaluated such a specific *repertoire* of adipokines in prepubertal age, our data are of interest because these chemokines represent crucial immune mediators involved in macrophage infiltration into adipose tissue, which reasonably occurred also in our cohort of obese children [5]. Moreover, as far as elevated concentrations of IL-8, MCP-1, IL-18 and MIF may precede the onset of T2D in adults [1], [33], [34], [35], our data indicate that, in prepubertal age, the association between these immune mediators and HOMA-IR appears basically explained by adiposity (supplementary Tables S3 and S4). Accordingly, in multivariate analysis, the inflammatory markers did neither affect the ability of leptin in predicting basal IR nor contributed significantly to variations in HOMA-IR (Table 4). On the other hand, the observation that the immune markers correlated directly with each other, also independently from individual's BMI (supplementary Table S4), suggests that sources other than fat tissue may contribute to the elevated circulating levels of these chemokines.

### Study limitations and Strengths

This study has some limitations that need to be considered. The cross-sectional nature of the survey does not allow conclusions to be drawn about the true prognostic values and the molecular mechanisms underlying the relationships between adipokines and IR. In addition, since in prepubertal age overweight and obesity may not necessarily track into adulthood, the prognostic relevance of these results should be further ascertained. However, obesity-linked variations of adipokines levels we found were almost comparable to those that predicted the risk of diabetes in adults [8], [13], [18], and the prognostic role of leptin also as a predictor of weight excess reduction was earlier demonstrated [29], [30]. Moreover, we did not characterize the middle- and low-molecular weight adiponectin oligomers, which may also have a differential distribution according to adiposity degree. On the other hand, the HMW multimers appear the major active forms in mediating the metabolic effects of adiponectin even in young children [9], [10], [11].

These constraints have to be weighed against the strengths of our survey which reside in the sample size, the representative nature of the population studied, the extensive characterization of the study participants, the simultaneous determination of multiple adipokines as well as the careful adjustment for various confounders using multivariable methods.

### Conclusions and Perspectives

In conclusion, our findings demonstrate that, as early as in prepubertal age, adiposity exhibits an unfavourable pattern of adipokines with minor sex-associated differences. However, among the adipose-derived mediators, leptin can be envisioned as a sex-independent discrimination marker of adiposity degree and, at least in girls, a reliable indicator of the systemic insulin resistance as estimated by HOMA-IR. Therefore, in prepubertal children, hyperleptinemia may allow the early identification of “at-risk” individuals, providing important prognostic information in predicting the impairment of glucose homeostasis.

## Supporting Information

Table S1 Spearman's rho correlation coefficients (r*_s_*) of leptin, HMW and inflammatory molecules with sex steroids in boys and girls.
(DOC)

Table S2 Spearman's rho correlation coefficients (r*_s_*) of leptin, HMW adiponectin and their ratio (L/HMW) with adiposity and insulin resistance measures, triglycerides and sICAM-1 in the combined study population.
(DOC)

Table S3 Spearman's rho correlation coefficients (r*_s_*) of leptin, HMW adiponectin, L/HMW and pro-inflammatory adipokines in the combined study population.
(DOC)

Table S4 Partial correlation coefficients of leptin, HMW adiponectin, L/HMW, pro-inflammatory adipokines and insulin-resistance measures after adjustment for BMI*_z-score._*
(DOC)

Table S5 Multiple regression models for the prediction of leptin (dependent variable) in boys. β-coefficients, *p-values* and determination coefficients of regression models (R^2^) are given.
(DOC)

Table S6 Multiple regression models for the prediction of leptin (dependent variable) in girls. β-coefficients, *p-values* and determination coefficients of regression models (R^2^) are given.
(DOC)
