# Supplementary material for: Inflammatory Adipokines, High Molecular Weight Adiponectin, and Insulin Resistance: A Population-Based Survey in Prepubertal Schoolchildren
Source: PLoS One. 2011 Feb 18;6(2):e17264. doi: 10.1371/journal.pone.0017264 (PMC3041818; doi:10.1371/journal.pone.0017264)
Supplement: Table S1 — Spearman's rho correlation coefficients (rs) of leptin, HMW and inflammatory molecules with sex steroids in boys and girls. (DOC) [file pone.0017264.s001.doc]

**Table S1**

|  |  | Leptin | HMW | IL-8 | IL-18 | MCP-1 | RANTES | MIF | sICAM-1 | Resistin |
| --- | --- | --- | --- | --- | --- | --- | --- | --- | --- | --- |
| **Boys** | Estradiol | *NA* | *NA* | *NA* | *NA* | *NA* | *NA* | *NA* | *NA* | *NA* |
|  | Free- Testosterone | 0.150 | 0.077 | -0.044 | 0.083 | -0.157 | -0.096 | 0.083 | -0.143 | 0.062 |
|  | Total-Testosterone | -0.085 | -0.042 | 0.071 | -0.071 | -0.092 | 0.132 | 0.061 | -0.037 | 0.051 |
| **Girls** | Estradiol | -0.124 | 0.163 | -0.106 | -0.021 | 0.034 | -0.009 | -0.180 | -0.011 | -0.080 |
|  | Free- Testosterone | 0.201 | -0.181 | -0.148 | -0.004 | -0.106 | 0.062 | 0.109 | 0.037 | 0.080 |
|  | Total-Testosterone | -0.044 | -0.036 | -0.019 | 0.107 | 0.101 | 0.094 | 0.049 | 0.108 | -0.047 |

Spearman’s rho correlation coefficients (r*_s_*) of leptin, HMW and inflammatory molecules with sex steroids in boys and girls.

*NA, not assessed*
