# Supplementary material for: Inflammatory Adipokines, High Molecular Weight Adiponectin, and Insulin Resistance: A Population-Based Survey in Prepubertal Schoolchildren
Source: PLoS One. 2011 Feb 18;6(2):e17264. doi: 10.1371/journal.pone.0017264 (PMC3041818; doi:10.1371/journal.pone.0017264)
Supplement: Table S2 — Spearman's rho correlation coefficients (rs) of leptin, HMW adiponectin and their ratio (L/HMW) with adiposity and insulin resistance measures, triglycerides and sICAM-1 in the combined study population. (DOC) [file pone.0017264.s002.doc]

**Table S2**

|  | BMI*_z-score_* | Fat Mass | Waist | Fasting  Insulin | HOMA-IR | Triglycerides | sICAM-1 | |
| --- | --- | --- | --- | --- | --- | --- | --- | --- |
| Leptin | 0.803***** | 0.797***** | 0.811***** | 0.602***** | 0.572***** | 0.424***** | 0.146**^†^** | |
| HMW | -0.216***** | -0.130**^‡^** | -0.237***** | -0.259***** | -0.227***** | **-**0.202**^§^** | -0.125**^‡^** | |
| Leptin/HMW | 0.756***** | 0.723***** | 0.781***** | 0.589***** | 0.548***** | 0.433***** | 0.180**^¶^** | |
|  | | | | | | | |  |

Spearman’s rho correlation coefficients (rs) of leptin, HMW adiponectin and their ratio (L/HMW) with adiposity and insulin resistance measures, triglycerides and sICAM-1 in the combined study population.

******p<*0.0001; **†***p*=0.01; **‡***p*<0.05; **§***p=*0.001 **^¶^***p<*0.01
