# Supplementary material for: Inflammatory Adipokines, High Molecular Weight Adiponectin, and Insulin Resistance: A Population-Based Survey in Prepubertal Schoolchildren
Source: PLoS One. 2011 Feb 18;6(2):e17264. doi: 10.1371/journal.pone.0017264 (PMC3041818; doi:10.1371/journal.pone.0017264)
Supplement: Table S3 — Spearman's rho correlation coefficients (rs) of leptin, HMW adiponectin, L/HMW and pro-inflammatory adipokines in the combined study population. (DOC) [file pone.0017264.s003.doc]

**Table S3**

|  | Leptin | HMW | L/HMW | Fasting Insulin | HOMA-IR | IL-8 | IL-18 | MCP-1 | RANTES | MIF | sICAM-1 | IP-10 | Resistin |
| --- | --- | --- | --- | --- | --- | --- | --- | --- | --- | --- | --- | --- | --- |
| Leptin | - | -0.192**^*^** | 0.888**^*^** | 0.602**^‡^** | 0.574**^‡^** | 0.018**^†^** | 0.030 | 0.191**^*^** | 0.233**^*^** | 0.207**^‡^** | 0.146**^†^** | 0.118**^†^** | 0.109 |
| HMW | -0.192**^*^** | - | -0.587**^‡^** | -0.259**^‡^** | -0.231**^‡^** | -0.086 | -0.085 | -0.063 | -0.049 | -0.066 | -0.127**^†^** | -0.105 | -0.027 |
| L/HMW | 0.888**^*^** | -0.587**^‡^** | - | 0.589**^‡^** | 0.552**^‡^** | 0.126**^†^** | 0.049 | 0.178**^§^** | 0.217 | 0.205 | 0.180**^§^** | 0.128**^†^** | 0.107 |
| Fasting Insulin | 0.602**^‡^** | -0.259**^‡^** | 0.589**^‡^** | - | 0.978**^‡^** | -0.081 | 0.010 | 0.158**^§^** | 0.184**^§^** | 0.189**^§^** | 0.252**^‡^** | 0.148**^†^** | 0.062 |
| HOMA-IR | 0.574**^‡^** | -0.231**^‡^** | 0.552**^‡^** | 0.978**^‡^** | - | -0.098 | 0.003 | 0.161**^§^** | 0.157**^§^** | 0.150**^†^** | 0.251**^‡^** | 0.137**^†^** | 0.045 |
| IL-8 | 0.118**^†^** | -0.086 | 0.126**^†^** | -0.081 | -0.098 | - | 0.214 | 0.225**^‡^** | 0.228**^‡^** | 0.279**^‡^** | 0.066 | 0.134**^†^** | 0.241**^‡^** |
| IL-18 | 0.030 | -0.085 | 0.049 | 0.010 | 0.003 | 0.214**^‡^** | - | 0.231**^‡^** | -0.015 | 0.241**^‡^** | 0.257**^‡^** | 0.379**^‡^** | 0.181**^§^** |
| MCP-1 | 0.191**^*^** | -0.063 | 0.178**^§^** | 0.158**^§^** | 0.161**^§^** | 0.225**^‡^** | 0.231**^‡^** | - | 0.159**^‡^** | -0.046 | 0.320**^‡^** | 0.212**^‡^** | -0.042 |
| RANTES | 0.233**^*^** | -0.049 | 0.217**^‡^** | 0.184**^§^** | 0.157**^§^** | 0.228**^‡^** | -0.015 | 0.159**^§^** | - | 0.450**^‡^** | 0.298**^‡^** | 0.056 | 0.313**^‡^** |
| MIF | 0.207**^‡^** | -0.066 | 0.205**^‡^** | 0.189**^§^** | 0.150**^†^** | 0.279**^‡^** | 0.241**^‡^** | -0.046 | 0.450**^‡^** | - | 0.352**^‡^** | 0.211**^‡^** | 0.638**^‡^** |
| sICAM-1 | 0.146**^†^** | -0.127**^†^** | 0.180**^§^** | 0.252**^‡^** | 0.251**^‡^** | 0.066 | 0.257**^‡^** | 0.320**^‡^** | 0.298**^‡^** | 0.352**^‡^** | - | 0.342**^‡^** | 0.362**^‡^** |
| IP-10 | 0.119**^†^** | -0.105 | 0.128**^†^** | 0.148**^†^** | 0.137**^†^** | 0.134**^†^** | 0.379**^‡^** | 0.212**^‡^** | 0.056 | 0.211**^‡^** | 0.342**^‡^** | - | 0.196**^§^** |
| Resistin | 0.109 | -0.027 | 0.107 | 0.062 | 0.045 | 0.241**^‡^** | 0.181**^§^** | -0.042 | 0.313**^‡^** | 0.638**^‡^** | 0.362**^‡^** | 0.196**^§^** | - |

Spearman’s rho correlation coefficients (r*_s_*) of leptin, HMW adiponectin, L/HMW and pro-inflammatory adipokines in the combined study population.

******p* = 0.001**; †***p*<0.05; **‡***p*<0.0001; **§***p*<0.01
