# Supplementary material for: Inflammatory Adipokines, High Molecular Weight Adiponectin, and Insulin Resistance: A Population-Based Survey in Prepubertal Schoolchildren
Source: PLoS One. 2011 Feb 18;6(2):e17264. doi: 10.1371/journal.pone.0017264 (PMC3041818; doi:10.1371/journal.pone.0017264)
Supplement: Table S4 — Partial correlation coefficients of leptin, HMW adiponectin, L/HMW, pro-inflammatory adipokines and insulin-resistance measures after adjustment for BMIz-score. (DOC) [file pone.0017264.s004.doc]

**Table S4**

|  | Leptin | HMW | L/HMW | Fasting Insulin | HOMA-IR | IL-8 | IL-18 | MCP-1 | RANTES | MIF | sICAM-1 | IP-10 | Resistin |
| --- | --- | --- | --- | --- | --- | --- | --- | --- | --- | --- | --- | --- | --- |
| Leptin | - | -0.031 | 0.722**^*^** | 0.391***** | 0.371***** | 0.003 | -0.037 | -0.004 | 0.109 | 0.108 | 0.002 | 0.063 | 0.011 |
| HMW | -0.031 | - | -0.660**^*^** | -0.178**^†^** | -0.149**^†^** | -0.056 | -0.073 | -0.011 | -0.003 | -0.028 | -0.091 | -0.086 | 0.001 |
| L/HMW | 0.722**^*^** | -0.660**^*^** | - | 0.374***** | 0.338***** | 0.024 | 0.001 | -0.007 | 0.090 | 0.108 | 0.067 | 0.080 | 0. 016 |
| Fasting insulin | 0.391***** | -0.178**^†^** | 0.374***** | - | 0.973***** | -0.179**^†^** | 0.070 | 0.191**^§^** | 0.140**^‡^** | 0.100 | 0.066 | 0.031 | -0.002 |
| HOMA-IR | 0.371***** | -0.149**^†^** | 0.338***** | 0.973***** | - | -0.190**^†^** | -0.033 | -0.054 | 0.067 | 0.075 | 0.191**^†^** | 0.103 | 0.018 |
| IL-8 | 0.003 | -0.056 | 0.024 | -0.179**^†^** | -0.190**^‡^** | - | 0.206**^§^** | 0.197**^§^** | 0.203**^§^** | 0.259**^*^** | 0.039 | 0.120**^‡^** | 0.226**^*^** |
| IL-18 | -0.037 | -0.073 | 0.001 | 0.070 | -0.033 | 0.206**^§^** | - | 0.222**^*^** | -0.029 | 0.232**^*^** | 0.250**^*^** | 0.375**^*^** | 0.174**^§^** |
| MCP-1 | -0.004 | -0.011 | -0.007 | 0.191**^§^** | -0.054 | 0.197**^§^** | 0.222**^*^** | - | 0.113**^‡^** | -0.094 | 0.289**^*^** | 0.194**^§^** | -0.076 |
| RANTES | 0.109 | -0.003 | 0.090 | 0.140**^‡^** | 0.067 | 0.203**^§^** | -0.029 | 0.113**^‡^** | - | 0.428**^*^** | 0.269**^*^** | 0.036 | 0.295**^*^** |
| MIF | 0.108 | -0.028 | 0.108 | 0.100 | 0.075 | 0.259**^*^** | 0.232**^*^** | -0.094 | 0.428**^*^** | - | 0.329**^‡^** | 0.197**^§^** | 0.630**^*^** |
| sICAM-1 | 0.002 | -0.091 | 0.067 | 0.066 | 0.191**^†^** | 0.039 | 0.250**^*^** | 0.289**^*^** | 0.269**^*^** | 0.329**^*^** | - | 0.332**^*^** | 0.346**^*^** |
| IP-10 | 0.063 | -0.086 | 0.080 | 0.031 | 0.103 | 0.120**^‡^** | 0.375**^‡^** | 0.194**^§^** | 0.036 | 0.197**^§^** | 0.332**^*^** | - | 0.187**^§^** |
| Resistin | 0.011 | 0.001 | 0. 016 | -0.002 | 0.018 | 0.226**^*^** | 0.174**^§^** | -0.076 | 0.295**^*^** | 0.630**^*^** | 0.346**^*^** | 0.187**^§^** | - |

Partial correlation coefficients of leptin, HMW adiponectin, L/HMW, pro-inflammatory adipokines and insulin-resistance measures after adjustment for BMI*_z-score._*

******p*<0.0001; **†***p*<0.01; **‡***p*<0.05; **§***p*<0.001
