# Supplementary material for: Inflammatory Adipokines, High Molecular Weight Adiponectin, and Insulin Resistance: A Population-Based Survey in Prepubertal Schoolchildren
Source: PLoS One. 2011 Feb 18;6(2):e17264. doi: 10.1371/journal.pone.0017264 (PMC3041818; doi:10.1371/journal.pone.0017264)
Supplement: Table S6 — Multiple regression models for the prediction of leptin (dependent variable) in girls. β-coefficients, p-values and determination coefficients of regression models (R2) are given. (DOC) [file pone.0017264.s006.doc]

**Table S6**

| Model | Independent  variable(s) | ß | *p-value* | Model R^2^ |
| --- | --- | --- | --- | --- |
|  |  |  |  |  |
| **1** | BMI*_z-score_* | 0.817 | <0.0001 | 0.668 |
| **2** | BMI*_z-score_* | 0.653 | <0.0001 | 0.735 |
|  | HOMA-IR | 0.245 | <0.0001 |  |
|  | Triglycerides | 0.112 | <0.05 |  |
|  | LDL | -0.038 | *NS* |  |
| **3** | BMI*_z-score_* | 0.675 | <0.0001 | 0.737 |
|  | HOMA-IR | 0.253 | <0.0001 |  |
|  | Triglycerides | 0.110 | <0.01 |  |
|  | LDL | -0.036 | *NS* |  |
|  | HMW | 0.031 | *NS* |  |
|  | MCP-1 | -0.047 | *NS* |  |
|  | RANTES | 0.005 | *NS* |  |
|  | MIF | -0.011 | *NS* |  |

Multiple regression models for the prediction of leptin (dependent variable) in girls. ß-coefficients, *p*-*values* and determination coefficients of regression models (R^2^) are given.

*NS,* statistically not significant.
